# Supplementary material for: Empirical methods for controlling false positives and estimating confidence in ChIP-Seq peaks
Source: BMC Bioinformatics. 2008 Dec 5;9:523. doi: 10.1186/1471-2105-9-523 (PMC2628906; doi:10.1186/1471-2105-9-523)
Supplement: Additional File 1 — A variety of html documents from the USeq web site detailing the available applications, their best usage, output file type descriptions, command line menus, etc. [file 1471-2105-9-523-S1.zip › USeqUserGuides/JavaDoc.zip!/JavaDoc/util/gen/package-use.html]

Uses of Package util.gen


|  |  |  |
| --- | --- | --- |
| **Overview** \| **Package** \| Class \| **Use** \| **Tree** \| **Deprecated** \| **Index** \| **Help** | |  |
| PREV   NEXT | **FRAMES**    **NO FRAMES**     **All Classes** |  |


---


## **Uses of Package util.gen**

| Packages that use util.gen | |
| --- | --- |
| **bioroot** |  |
| **cgar** |  |
| **cgar.beans** |  |
| **expr** | Classes and tools related to expression array analysis. |
| **trans.cel** | Apps and methods for manipulating cel files. |
| **trans.graphics** | For graphically representing '.cel' files and intervals. |
| **util.gen** | Variety of helper methods for Email, IO, Encryption, HTML, Swing... |

Classes in util.gen used by bioroot

****Email****

Classes in util.gen used by cgar

****SQL****
  
          Utility class to hold database and relevant fields and methods, some from Hall and Brown,
most from BioRoot.

Classes in util.gen used by cgar.beans

****SQL****
  
          Utility class to hold database and relevant fields and methods, some from Hall and Brown,
most from BioRoot.

Classes in util.gen used by expr

****RankedFloatArray****

Classes in util.gen used by trans.cel

****CubicSpline****
  
          For calculating cubic splines of incremental data, interpolation, from http://www.frank-buss.de/CubicSpline.java

****LinearRegression****
  
          For calculating a simple linear regression.

Classes in util.gen used by trans.graphics

****TextFrame****
  
          Creates a free standing plain editable text window.

Classes in util.gen used by util.gen

****Histogram****
  
          Generates histograms.

****RankedFloatArray****

****RankSample****

---


|  |  |  |
| --- | --- | --- |
| **Overview** \| **Package** \| Class \| **Use** \| **Tree** \| **Deprecated** \| **Index** \| **Help** | |  |
| PREV   NEXT | **FRAMES**    **NO FRAMES**     **All Classes** |  |


---
